# TACKoMesh – A randomised controlled trial comparing absorbable versus non-absorbable tack fixation in laparoscopic IPOM + repair of primary incisional hernia using post-operative pain and quality of life - Reliatack™ versus Protack™

**DOI:** 10.1007/s10029-024-03111-y

**Published:** 2024-08-23

**Authors:** J. James Pilkington, James Pritchett, Catherine Fullwood, Annie Herring, Fiona L. Wilkinson, Aali Jan Sheen

**Affiliations:** 1 Department of Life Sciences, Faculty of Science and Engineering, Manchester Metropolitan University, John Dalton Building, Chester Street, Manchester, UK; 2 Department of Hernia Surgery, Manchester University NHS Foundation Trust, Manchester, UK; 3 Centre for Biostatistics, Division of Population Health, Health Services Research & Primary Care, School of Health Sciences, Faculty of Biology, Medicine and Health, University of Manchester, Manchester, UK; 4 Research and Innovation, Manchester University NHS Foundation Trust, Manchester, UK

**Keywords:** Incisional hernia, Mesh-fixation, Pain, PROMs, Intraperitoneal onlay mesh (IPOM), Laparoscopic surgery

## Abstract

There is a clinical need to better understand and improve post-operative pain for patients undergoing laparoscopic repair of incisional hernia. The aim of this single-centre, double-blind, randomised controlled trial was to compare post-operative pain between absorbable and non-absorbable tack fixation in patients undergoing IPOM + repair. Patients with primary incisional hernia (size 3–10 cm), were randomised to either Reliatack™ (*n* = 27), an articulating-arm device deploying absorbable polymer tacks, or Protack™ (*n* = 36), a straight-arm device deploying permanent titanium tacks. The primary outcome was reported pain on activity using a visual analogue scale at post-operative day 30. Clinical and patient-reported outcome measures (PROMs) were assessed pre-operatively (day 0), and at 1-, 6-, 30- and 365-days post-surgery. No significant differences in reported pain ‘on activity’ were found at any timepoint. Less reported pain ‘at rest’ was found on post-operative day-1 with absorbable tacks (*p* = 0.020). Significantly longer mesh-fixation time (*p* < 0.001) and the use of more knots for fascial closure (*p* = 0.006) and tacks for mesh-fixation (*p* = 0.001) were found for the absorbable tack group. There were no differences in other clinical and PROMs between groups. For the whole trial cohort (*n* = 63) several domains in the Short-Form-36 showed a reduction from baseline scores at day 30 that improved at day 365. At post-operative day 30, 75.0% of patients reported ‘a lot of pain’ since discharge. This study found no difference in reported pain when choosing absorbable or non-absorbable tack fixation. The utility of “early” post-operative pain assessment as a comparator following incisional hernia repair needs clarification.

**Supplementary Information:**

The online version contains supplementary material available at 10.1007/s10029-024-03111-y.

## Introduction

Incisional hernia incidence increases from 12.6% at 1 year to 22.4% at 3 years post-surgery [9] and is associated with significant psychosocial morbidity [28] and healthcare costs [10]. Laparoscopic intraperitonal onlay mesh (IPOM) repair remains a recommended option for treatment of incisional hernia with a defect size of 2–6 cm [14]. Abdominal pain is the most common reason for representation to services following laparoscopic incisional hernia repair [14]. The use of pain as a comparator following incisional hernia repair techniques has not yet been standardised.

European registry data from Denmark showed a greater use of laparoscopic compared to open incisional hernia repair from 2007 to 2018 (57.5% vs. 42.5%) [14]. Data from Germany has shown a more recent trend away from the use of laparoscopic intraperitoneal onlay mesh (IPOM) repairs (33.8% in 2013 to 21% in 2019) [17]. Reporting of rare but serious complications, including bowel compromise and mesh migration, has played a role in this trend [18]. A hernia research group has identified long-term follow-up on patients that underwent IPOM repair as a research priority [24].

Decision making in recommending treatment options for incisional hernia is complex and must take into account patient and hernia factors, e.g. BMI and size of defect, alongside complication profiles. For the available options, different complication profiles have been shown; for example in laparoscopic vs. open repairs the rates of readmission and reoperation are lower [14]. Failure in the use of a standardised outcome set and under-reporting of patient-reported outcome measures (PROMs) has been highlighted in the existing evidence base when comparing incisional hernia repair techniques [12]. Balancing risks and patient wishes are important in the consent for incisional hernia repair and a greater understanding of pain following incisional hernia repair will facilitate these discussions.

The IPOM + technique includes sutured closure of the fascial defect prior to intraperitoneal mesh placement [26]. Sutured fascial closure within the laparoscopic repair has shown benefits in reducing chronic pain, seroma formation and poor cosmesis [15].

Minimum datasets for reporting on incisional hernia trials have been published. They recommend ‘pain at rest’, ‘pain on activity’ and ‘pain felt during the last week’ be assessed [21]. They do not give an indication on the best and most appropriate timing in which these assessments take place [1, 16, 19]. Serial pain assessment, key clinical outcomes and PROMs were collected pre-operatively and at four post-operative timepoints up to one year [25]. The primary outcome, reported pain at day 30 following surgery, was chosen as the use of absorbable vs non-absorbable tacks has been thought to affect the early- to mid-term post-operative pain experienced by patients. This is in the window of time in which patients present to hospital services with abdominal pain following laparoscopic repair [14].

### Study aim

To establish a difference in post -operative pain between a choice of two spiral tack mesh-fixation devices when used to perform laparoscopic incisional hernia repair by the IPOM + technique.

### Objectives

To compare post-operative pain scores between absorbable and non-absorbable tack fixation, measured using a visual analogue scale (0–10 cm), at post-operative day 30 following laparoscopic IPOM + repair.

To compare post-operative clinical outcomes between absorbable and non-absorbable tack fixation.

To compare patient-reported outcomes between absorbable and non-absorbable tack fixation.

## Methods

### Participants

TACKoMesh is a prospective, single-centre, double-blind parallel RCT (NCT03434301). Participants with primary incisional hernia, with defect size 3–10 cm and a minimum distance of 3 cm from costal margin or pelvic brim, underwent elective laparoscopic incisional hernia repair. For full eligibility criteria see published trial protocol [25]. Exclusion criteria included age < 18 or > 80 years old, previous attempt at repair, BMI > 40 kg/m^2^ and inability to close the defect during surgery [25].

Screening took place in specialist outpatient clinics at MFT. Following consent for laparoscopic incisional hernia repair, patients were provided with trial information ahead of recruitment into the trial (Research Ethics Committee reference 17/NW/0082; Integrated Research Application System project ID 213,428).

### Treatment allocation

Patients were randomised to surgery with Reliatack™ (Medtronic, Medtronic.com), an articulating device deploying absorbable plastic copolymer tacks, or Protack™ (Medtronic, Medtronic.com), a straight arm device deploying non-absorbable tacks on the day of surgery using a sealed envelope technique that stratified patients according to size of hernia defect (3–6 cm or > 6–10 cm). Sealed envelopes were generated prior to trial initiation by an independent member of the trial team.

Patients were enrolled by the blinded researcher (JJP) and principal investigator (AJS). Randomisation was performed in theatre by the principal investigator (AJS). Defect size had been recorded in clinic with the use of available cross-sectional imaging or, where imaging was unavailable, clinical examination in the supine position by two independent examiners (JJP & AJS). Intra-operative details were recorded at the end of the procedure. Patients were followed up by face-to-face encounter at post-operative days 1, 6, 30, and 365 by the blinded researcher (JJP) in the inpatient and outpatient setting. Excluding the operating surgeon (AJS), all patients and members of the trials team were blind to treatment allocation throughout trial conduct and data management up unto unblinding for analysis.

### Surgical procedure

Following pneumoperitoneum, adhesiolysis and fascial closure, Symbotex TM (Medtronic, Medtronic.com) mesh was secured by either absorbable or non-absorbable tack fixation.

All cases were performed by AJS [25]. Skin preparation and antimicrobial prophylaxis was standardised and recorded. Establishing pneumoperitoneum and adhesiolysis was performed at intrabdominal pressure 12-15mmHg. Fascial closure was performed using a 1 − 0 Loop Maxon with one end cut to provide extra length and an extracorporeal knot slid down using an Endoloop pusher at intrabdominal pressure 8-10mmHg. Interrupted sutures were sited sequentially to close the defect and the number of knots used was recorded in the operative data case report form. Sizing of the mesh took place, appropriate to achieve mesh overlap of 3 cm in all directions. This was followed by mesh placement and fixation with the allocated mesh-fixation device with tacks deployed in a double-crown arrangement and without the use of transfascial sutures [25]. At the end of the procedure, a recorded amount of local anaesthetic was infiltrated as a bilateral TAP block under direct visualisation with the laparoscope in addition to infiltration at port sites.

### Outcomes

The primary outcome of the trial was reported pain on activity, at 30 days following surgery. This was measured using a visual analogue scale (VAS)(0–10).

**Fig. 1:**
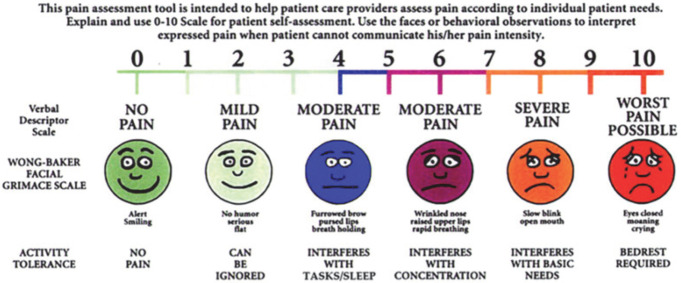
Universal Pain Assessment Tool (18)

To obtain pain scores, trial participants were shown the validated universal pain assessment tool (Fig. 1) [7]. The blinded researcher (JJP) then asked the following standardised questions; “Please indicate on this scale the pain that you currently experience from your incisional hernia **at rest**?” and “Please indicate on this scale the pain that you currently experience from your incisional hernia **during activity**?”.

Secondary objectives were to assess pain scores at rest and on activity on days 1, 6 and 365 post-surgery, and to compare key clinical and PROMs between treatment arms. A full list of secondary outcome measures can be found within the published trial protocol [25]. All pain scores were collected in the same manner as for the primary outcome. The operative data recorded included details on mesh size, number of tacks used, number of knots used, time to perform mesh fixation (recorded as the time from deploying first to last spiral tack) and total length of surgery. Key clinical outcomes and complications were collected in trial specific case report forms with the same blinded researcher at face-to-face patient reviews which included a clinical examination. Complications were subsequently graded as per the Clavien-Dindo classification [6]. Additional PROMs were obtained via validated questionnaires, the Short Form 36 (SF-36) and Carolina Comfort Score (CCS), and a trial-specific questionnaire designed for the TACKoMesh trial that included standard questions that have been used in other comparative trials and were approved by the local ethics and trial steering committee. The SF-36 was issued at three timepoints; pre-operative day 0, and post-operative days 30 and 365. The CCS was issued at two post-operative time points; day 30 and day 365. The TACKoMesh PROMs questionnaire was issued once at post-operative day 30. It included non-validated questions on trial participants’ pain experience and recovery to normal activity since discharge from the hospital.

### Sample size

A power calculation was performed using similar studies as a reference [3–5; 8]. A change in VAS score of 1.5 was deemed to be clinically significant based on the interpretation of previous results and timing of pain assessments. A sample size of 74 patients was required to detect a significant difference of 1.5 in VAS pain score between treatment groups with 80% power, and alpha of 0.05, SD of 2 and 20% drop out.

### Statistics

Statistical analysis was performed in RStudio v1.4.1106. For comparisons between groups, t-test, Wilcoxon Rank Sum test, and Fisher’s Exact test were used based upon the type of data and its distribution. In cases where repeat data were collected at multiple timepoints, and the assumptions were valid, repeated measures ANOVA was used. Where missing data was encountered, the patient was removed from that particular analysis, with the exception of SF-36 and CCS data, where validated instructions on imputation of missing data were followed.

## Results

### Demographics

**Fig. 2:**
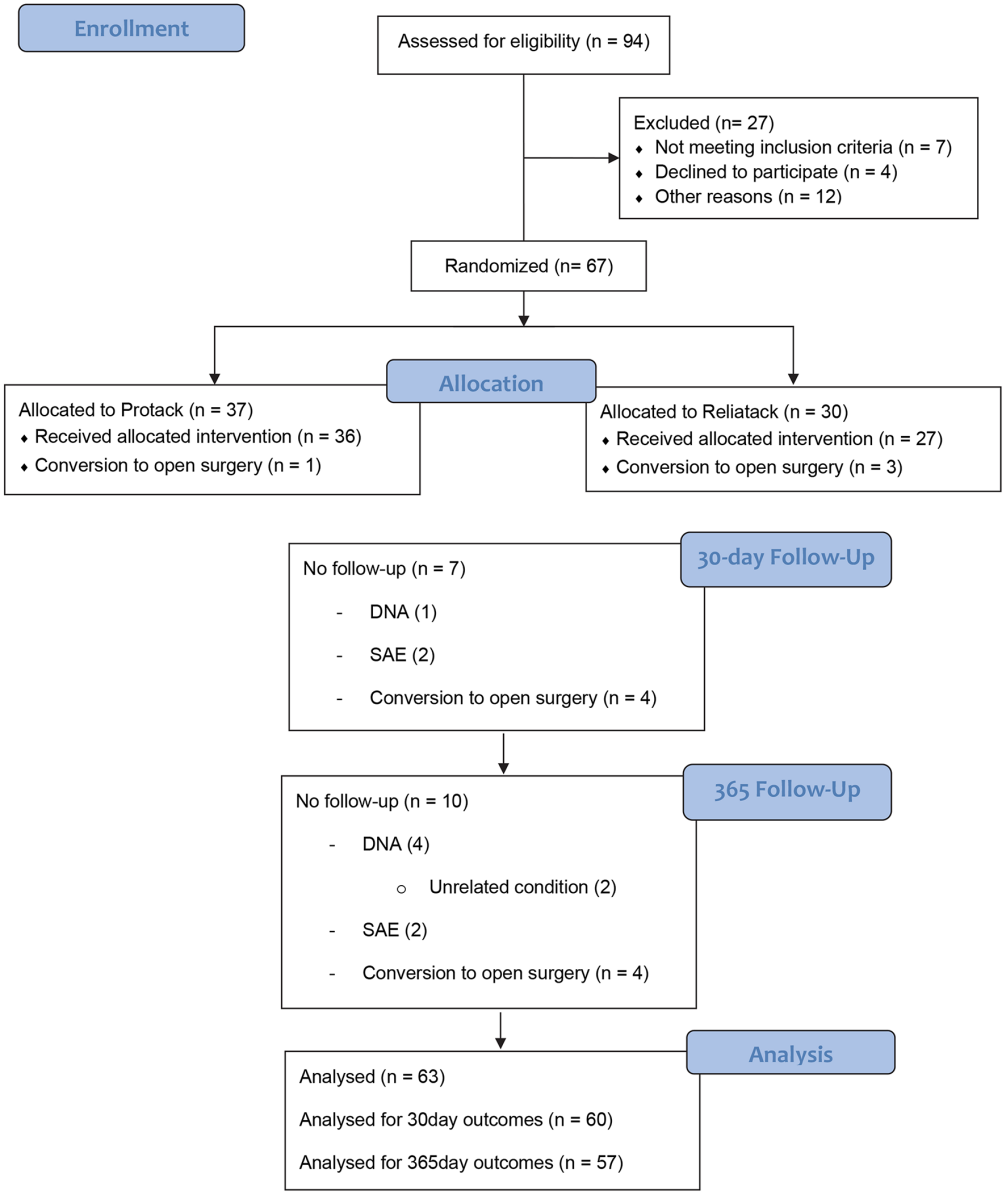
CONSORT diagram for TACKoMesh RCT

Sixty-seven patients were randomised to treatment with absorbable (Reliatack™)(*n* = 30) or non-absorbable (Protack™)(*n* = 37) tack fixation (Fig. 2). Four patients were converted to open retro-rectus repair due to dense intraabdominal adhesions and patient safety considerations for proceeding laparoscopically; one experienced an iatrogenic small bowel injury. They were removed from ongoing follow-up as they did not receive either method of spiral tack mesh-fixation. Recruitment began in July 2017 and surgeries took place between July 2017 and March 2020.

Patient groups were evenly matched on baseline demographics (Table 1) and proposed risk factors for incisional hernia formation in all areas except age [23] (Appendix 1).

**Table 1:** Baseline patient, Pain, and hernia demographics. P-values obtained from ∞ T-test, ∆ wilcoxon rank Sum Test, and † Fisher’s exact test based upon the type of data and its distribution

	Entire cohort ( *N* = 63 )	Protack ( *N* = 36 )	Reliatack ( *N* = 27 )
**Age (years)**,** mean (SD)**	59.1 (12.6)	57.3 (11.4)	61.6 (13.9)
**Male gender**,** n (%)**	36 (57.1%)	20 (55.6%)	16 (59.3%)
**BMI**,** mean (SD)** (N)	30.91 (5.11) (62)	31.90 (5.44) (36)	29.52 (4.35) (26)
**Diabetic (n (%))** - Diet & lifestyle - Tablet control - Insulin therapy	10 (15.9%) - 1 (1.6%) - 4 (6.4%) - 5 (7.9%)	4 (11.1%) - 0 (0.0%) - 2 (5.6%) - 2 (5.6%)	6 (22.2%) - 1 (3.7%) - 2 (7.4%) - 3 (11.1%)
**Smoking history (n (%))** - Current smoker - Ex-smoker	32 (50.8%) - 6 - 26	16 (44.4%) - 3 - 13	16 (59.3%) - 3 - 13
**On daily analgesics**,** n (%)**	7 (11.1%)	4 (11.1%)	3 (11.1%)
**Defect size in clinic**,** median [IQR]**	5.0 [4.0 to 7.0]	5.0 [4.0 to 7.0]	5.0 [4.0 to 7.5]
**Index laparotomy**,** n (%)** - Midline - Lower midline - Lanz/Gridion - Rooftop/Kocher/Subcostal - Flank - Pfannestiel - Rutherford Morrison - Previous congenital hernia - Lap. port site - Other - Missing	19 (30.2%) 3 (4.8%) 1 (1.6%) 20 (31.8%) 2 (3.2%) 3 (4.8%) 0 1 (1.6%) 3 (4.8%) 10 (15.9%) 1 (1.6%)	10 (27.8%) 2 (5.6%) 0 11 (30.6%) 1 (2.8%) 2 (5.6%) 0 (0.0%) 1 (2.8%) 2 (5.6%) 6 (16.7%) 1 (2.8%)	9 (33.3%) 1 (3.7%) 1 (3.7%) 9 (33.3%) 1 (3.7%) 1 (3.7%) 0 (0.0%) 0 (0.0%) 1 (3.7%) 4 (14.8%) 0 (0.0%)
**Short form 36 domain scores**,** median [IQR]** - Physical function - Physical health - Emotional health - Energy/fatigue - Emotional well being - Social function - Pain - General health	70.0 [36.0–85.0] 25.0 [0.0-100.0] 66.7 [0.0-100.0] 55.0 [43.8–65.0] 76.0 [52.0–84.0] 75.0[50.0-100.0] 57.5 [45.0-77.5] 59.4 [50.0-68.8]	70.0 [33.8–85.0] 25.0 [0.0-87.5] 100 [33.3–100] 57.5 [41.3–65.0] 74.0 [58.0–83.0] 75.0 [50.0-87.5] 45.0 [24.4–67.5] 62.5 [43.8–68.8]	65.0 [41.3–91.3] 50.0 [0.0-100.0] 66.7 [0.0-100.0] 55.0 [45.0–65.0] 76.0 [52.0–84.0] 81.3 [62.5–100.0] 57.5 [45.0–80.0] 56.3 [50.0-68.8]

Response rate for the primary outcome at 30 days was 95.5%. Other case report form response rates varied from 87.3 to 100.0%. For the SF-36, response rates at days 0, 30 and 365 were 73.0%, 90.5% and 85.7% respectively. For the CCS, response rates on days 30 and 365 were 92.1% and 85.7% respectively. All available data was included in analysis.

The trial was running at the time of the COVID-19 outbreak. Prolonged reduced access to elective operating theatre space within the NHS led to early closure of trial. A small number of patients were undergoing trial follow-up at the time of the coronavirus lockdown. For these patients, available primary and secondary outcome measures were collected via telephone. Those outcomes requiring face-to-face contact were delayed until the lifting of restrictions. Follow up took place until March 2021.

### VAS pain scores: absorbable (Reliatack™) vs. non-absorbable (Protack™)

**Fig. 3:**
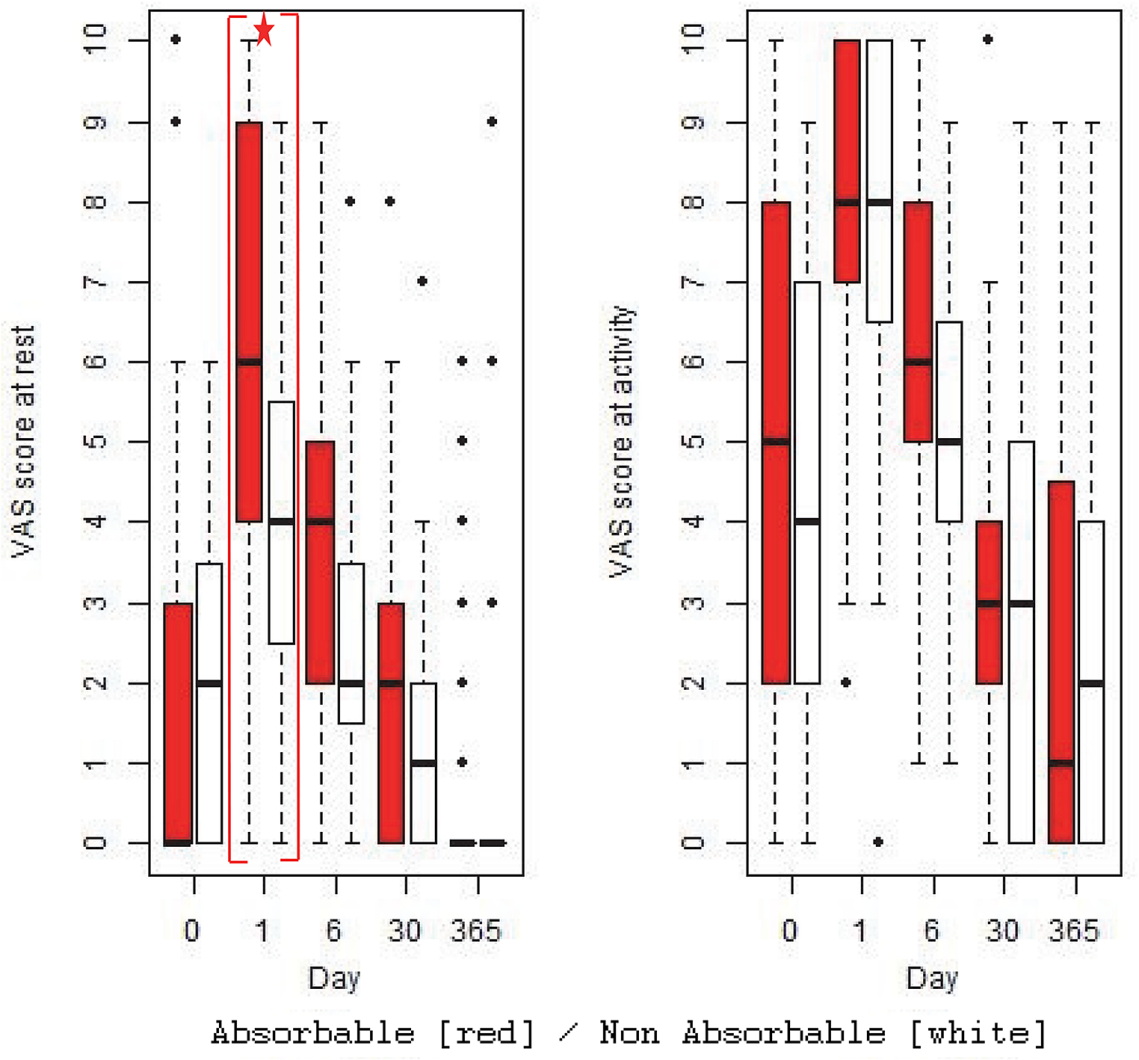
‘VAS Pain Scores at Rest’ and ‘VAS Pain Scores on Activity’ at All Trial Timepoints (Day) for patients separated by Treatment Arm. Boxplots showing median (bold line), interquartile range (box), values within 1.5x IQR (whiskers) and outliers (dots) for patients separated by treatment arm; Protack™ [Red] versus Reliatack™ [White]. Where the difference in reported pain scores was significant between the groups (*p* < 0.05) this is indicated by red brackets and a star

There was no significant difference in VAS pain score on activity at any timepoint between the two treatment arms (Fig. 3). There was significantly less reported pain at rest on post-operative day 1 with absorbable tacks (*p* = 0.020), with a diminishing trend towards less pain at 6- and 30-days post-surgery which did not reach statistical significance (Fig. 3). There were no significant differences between groups in the use of patient-controlled analgesia or analgesic regime on 1-, 6- or 30-days post-surgery.

### Additional outcome measures: absorbable (Reliatack™) vs. non-absorbable (Protack™)

A summary of operative data is supplied in Table 2. All procedures were recorded as ‘Clean’, and the volume of predicted blood loss was comparable between the groups.

**Table 2:** Operative Data. P-values obtained from ∞ T-test, ∆ wilcoxon rank Sum Test, and † Fisher’s exact test based upon the type of data and its distribution

	Entire cohort ( *N* = 63 )	Protack ( *N* = 36 )	Reliatack ( *N* = 27 )	*p*-value
ASA grade, n (%) - ASA 1 - ASA 2 - ASA 3 - ASA 4 - missing	12 (19.0%) 41 (65.1%) 5 (7.9%) 0 (0.0%) 5 (7.9%)	6 (16.7%) 26 (72.2%) 1 (2.8%) 0 (0.0%) 3 (8.3%)	6 (22.2%)) 15 (55.6%) 4 (14.8%) 0 (0.0%) 2 (7.4%)	0.176 †
Intraoperative instability, n (%)	3 (4.8%)	3 (8.3%)	0 (0.0%)	0.254 †
Duration of surgery (minutes), median [IQR]	80.0 [55.0–92.5]	74.0 [48.8–90.0]	90.0 [60.0–97.5]	0.294 ∞
> 1 defect identified, n (%)	13 (20.6%)	8 (22.2%)	5 (18.5%)	0.764 †
Number of knots, median [IQR]	3.0 [2.0–3.5]	2.0 [1.0–3.0]	3.0 [2.5–4.0]	**0.006 ∆**
Chosen mesh size (n (%) - 12 cm round - 15 cm round - 15 × 20 cm - 20 × 25 cm - 30 × 20 cm - Other-	0 (0.0%) 5 (7.94%) 17 (26.98%) 20 (31.75%) 9 (14.29%) 6 (9.52%)	0 4 (11.11%) 12 (33.33%) 9 (25.00%) 4 (11.11%) 4 (11.11%)	0 1 (3.70%) 5 (18.52%) 11 (40.74%) 5 (18.52%) 4 (14.81%)	0.480 †
Number of tacks, median [IQR]	26.0 [22.0–28.5]	23.0 [20.0–27.0]	28.0 [26.0–30.0]	**0.001** ∆
Mesh-fixation time (secs), median [IQR]	286.0 [159.5–428.0]	171.0 [130.0–270.5]	430.0 [330.0–473.5]	**< 0.001** ∆

More knots (3 [2.5-4] vs. 2 [1–3]) and tacks (28 [26–30] vs. 23 [20–27]) were used and the time to perform mesh fixation (430 [330-473.5] vs. 171 [130-270.5] seconds) was longer in the absorbable tack group (*p* < 0.001). Length of hospital stay and incidence of hernia recurrence, seroma formation, surgical site infection, and Clavien-Dindo Grading of complications were comparable between treatment groups (Table 3). The SF-36, the TACKoMesh patient questionnaire and CCS questionnaire all showed no significant differences between groups (Appendix 2).

**Table 3:** Clinical and economic outcomes. P-values obtained from ∞ T-test, ∆ wilcoxon rank Sum Test, and † Fisher’s exact test based upon the type of data and its distribution

	Entire cohort (*N* = 63 )	Protack (*N* = 36 )	Reliatack (*N* = 27 )	*p*-value
**Length of stay (days)**,** median [IQR]** **(N)**	3.5 [2.0–6.0] (60)	3.0 [2.0–6.0] (34)	4.0 [2.0–5.8] (26)	0.809 ∆
**Hernia recurrence**,** n (%)**	8 (12.7%)	4 (11.1%)	4 (14.8%)	0.715 †
**Seroma formation**,** n (%)** - **Total** - **Type I** - **Type II** - **Type III** - **Type IV**	33 (52.4%) 9 16 3 5	17 (47.2%) 6 8 2 1	16 (59.3%) 3 8 1 4	0.446†
**Surgical site infection**,** n (%)**	7 (11.1%)	4 (11.1%)	3 (11.1%)	1.000 †
**Clavien-Dindo Grade 3 to 5 Complication**,** n (%)**	9 (14.3%)	4 (11.1%)	5 (18.5%)	0.480 †

### Hernia size as a covariate

As part of randomisation to treatment arm, patients were stratified based upon defect size: (i) 3–6 cm (*n* = 44) and, (ii) > 6–10 cm (*n* = 19). Patients with a hernia > 6–10 cm showed an increased rate of hernia recurrence (26.3% vs. 6.8%, *p* = 0.047) and seroma formation (73.7% vs. 43.2%, *p* = 0.031) and a larger proportion of higher classed seromas identified (*p* = 0.021) when compared to patients with a 3–6 cm hernia. No significant differences were found in reported pain scores at rest or on activity at all time points, total length of stay, and incidence of surgical site infection and Clavien-Dindo Grade III-IV complication.

### Clinical outcomes for the entire trial cohort

The reported VAS pain scores at rest and activity for the entire trial cohort (Appendix 3) showed an initial worsening in reported pain on post-operative day 1 that reduced at day 6 to near pre-operative levels, with improvement, for many, by day 30. By day 365 an overall improvement is seen in reported pain at rest and on activity by comparison to pre-operative levels for almost all patients.

Pain at rest at one year following surgery was reported by 7 (11.1%) patients. Patients with persistent pain at 1 year reported higher pain pre-operatively at rest (5 [2–7] vs. 0 [0–3] *p* = 0.018) and on activity (8.5 [5–10] vs. 4 [2–8] *p* = 0.018).

Sutured closure of the fascial defect was performed in all cases with complete closure obtained in 95.2% cases. More than one hernia defect was identified at laparoscopy in 13 (20.6%) cases; with instances of up to four separate defects noted, a *‘Swiss cheese’* hernia [11]. There was one instance of two pieces of mesh being required and one instance of a piece being altered prior to placement. Adequate mesh overlap (> 3 cm) was achieved in all cases. In two cases, additional laparoscopic port placement was required to facilitate mesh siting.

9/63 (14.3%) patients experienced a Clavien-Dindo Grade III to V complication (Table 3). Two patients required ultrasound-guided seroma intervention with one going on to require an abdominal wall washout under general anaesthesia. Five patients required an unplanned admission to critical care for respiratory support in the post-operative period. Three patients required emergency surgery during one year of follow-up for (i) iatrogenic small bowel injury, (ii) incarcerated port site hernia, and (iii) incarcerated hernia recurrence. There was one 30-day mortality owing to pulmonary embolus. Grade 2 complications were surgical site infection, lower respiratory tract infection, urinary tract infection and secondary haemorrhage requiring transfusion. Grade 1 complications included urinary retention, paralytic ileus, and constipation.

For most domains of the SF-36, scores at post-operative day 30 were reduced compared to pre-operative scores. Scores at day 365 were higher (Appendix 4) These findings were most marked for the ‘Physical health’ and ‘Emotional health’ domains. ‘Emotional well-being’ and ‘General health’ were least influenced by surgery. Patients who reported persistent pain at day 365 tended to provide lower pre-operative QOL scores compared to patients with no pain following treatment. This was most marked for the ‘Social function’ domain (50 [31–50] vs. 75 [63–100] *p* = 0.006).

At day 30, patients were asked qualitative questions regarding their recovery (Appendix 5). In response to the ‘yes/no’ question, ‘Since your discharge from hospital did you experience a lot of pain?’, 45/60 (75.0%) patients responded ‘yes’ with 6 (10.0%) of these patients going on to select the option to describe the pain as ‘severe to unbearable’. Of other notable PROMs, it was ‘1–2 weeks’ before 50.0% of responders could ‘cook or clean’ and ‘walk without painkillers’, and it was ‘> 3 weeks’ before 84.6% and 78.4% of responders ‘returned to work’ and ‘report being fully recovered’ respectively.

## Discussion

The pain experienced following incisional hernia repair needs greater understanding to best facilitate patient selection and counselling. There was a vogue to using absorbable tacks at the time of study design with a suggestion that absorbable tacks cause less pain and are less likely to cause bowel or visceral injury. No difference in reported pain on activity at post-operative day 30 was found when making a choice between absorbable and non-absorbable tack fixation. This study provides data on the laparoscopic IPOM + repair with clinical and patient-reported outcomes. The study closed early owing to the outbreak of COVID-19, it is subsequently underpowered, and that is a key limitation of this study.

Consistency in outcomes reporting is needed for incisional hernia studies [22]. A standardised outcome set recommends assessing chronic pain at 3 months, 1 and 5 years but gave no recommendation on the timing of early post-operative pain assessment [21]. Abdominal pain is the most common reason for representation to healthcare services following laparoscopic incisional hernia repair [14] Within this trial there were no significant differences in reported pain ‘on activity’ between treatment arms at all post-operative time points up to one year. When comparing pain scores at rest, there was a trend towards less pain with absorbable tacks at all post-operative timepoints, and a significant difference at post-operative day 1. This observation, coupled with findings that patients in the absorbable tack group reported more pain pre-operatively and had more sutures and tacks used during surgery, may suggest some improvements in post-operative pain when choosing absorbable tacks. This study did not reach power and the finding needs further clarification.

Recent guidelines on reporting for incisional hernia trials suggest future assessors specify ‘pain at the hernia site’ and ask for a score on a scale of 0–10 for ‘pain at rest (lying down)’, ‘pain during activities (walking, cycling, sports)’, and ‘pain felt during the last week’ [21]. Pre-operative pain is known to be a predictor of post-operative pain and reduced quality of life following ventral hernia repair [27]. There is no current recommendation on recording pre-operative pain and its impact on subsequent pain assessment and analysis. Standardisation of pain assessments following incisional hernia repair techniques would greatly facilitate future comparisons [14]. Pain scoring seems multifactorial and consistency in reporting would enhance understanding of the patient experience and its utility in practice.

This study found that mesh-fixation time was significantly longer in the absorbable tack fixation group (430 [330-473.5] vs. 171 [130-270.5] seconds). This is likely to be a product of technical differences between the design of the two tacking devices. Reliatack™ has an articulating arm and reloadable tack cartridges, which leads to increased operating time articulating the device and increased scrub time reloading the cartridges.

Recurrence rates within this study are marginally higher than quoted in the literature [2]. At the design and initiation of this trial, guidelines on the laparoscopic repair of ventral hernia allowed for a hernia with a defect of 3–10 cm in size to undergo surgery with a need for mesh overlap of > 3 cm. More recent publications have seen guidance change to hernia with defect 2–6 cm [14] and mesh overlap to > 5 cm [2]. This study found a greater risk of incisional hernia recurrence with defects sized 6–10 cm compared with 3–6 cm (26.3% vs. 6.8%, *p* = 0.047). Supporting this recent update to guidance. 

The study found high rates of seroma formation with this technique. This could be a product of the chosen method of fascial closure, interrupted suturing. Alternatively, the increased rates of detection could be owing to trial follow-up conditions. The majority were asymptomatic, detected at an early trial visit and resolved without intervention. This study, like others, showed that persistent seromas and seromas requiring intervention present a risk of preceding infective complications and hernia recurrence [13, 20].

A benefit to laparoscopic methods of incisional hernia repair identified by this study was the ability of a surgeon to identify multiple hernia defects during laparoscopy. An additional opinion from this trial was the success of this technique in the treatment of often difficult to fix incisional hernia, e.g. flank hernia. It is also proposed that laparoscopic techniques require less abdominal wall dissection and distortion to native anatomy. This should afford a greater choice of surgical options for the repeat attempt at repair in the instance of hernia recurrence.

During the outbreak of COVID-19 and announcement that the UK was going into lockdown, three trial participants were inpatients following surgery and one patient had been operated on the week prior. This had an impact on the trial follow-up for these patients given that new restrictions were placed on patient contact for research purposes. Follow-up milestones were maintained for pain scores and other information that could be obtained via telephone consultation. The impact of COVID, particularly the early closure of the trial, is a key limitation.

It seems the IPOM + repair has fallen out of favour. As a treatment option for the hernia surgeon there is a sub-population of patients that might benefit from its outcome profile. Longer term data is needed for the IPOM + repair [24].

## Conclusion

This study found no difference in reported pain on activity following elective laparoscopic incisional hernia repair, with IPOM + repair, when choosing absorbable or non-absorbable tack fixation. The utility of “early” post-operative pain assessment as a comparator following incisional hernia repair needs clarification.

## Electronic supplementary material

Below is the link to the electronic supplementary material.

Supplementary Material 1

## Abbreviations

ANOVA: analysis of variance
BMI: Body mass index
CCS: Carolina Comfort Score
IPOM: Intraperitoneal onlay mesh
MFT: Manchester University NHS Foundation Trust
NHS: National Health Service
PROMs: Patient–reported outcome measures
QOL: Quality of life
RCT: Randomised controlled trial
SD: Standard deviation
SF–36: Short Form 36
VAS: Visual analogue scale
