# Supplementary material for: TACKoMesh – A randomised controlled trial comparing absorbable versus non-absorbable tack fixation in laparoscopic IPOM + repair of primary incisional hernia using post-operative pain and quality of life - Reliatack™ versus Protack™
Source: Hernia. 2024 Aug 23;28(5):1879–88. doi: 10.1007/s10029-024-03111-y (PMC11457574; doi:10.1007/s10029-024-03111-y)
Supplement: Supplementary file 1 — Supplementary Material 1 [file 10029_2024_3111_MOESM1_ESM.docx]

Appendix 1 - Distribution of Patient Risk Factors for Incisional Hernia Formation

*P-values obtained from* ∞ *T-test,* ∆ *Wilcoxon Rank Sum Test, and* † *Fisher’s Exact Test based upon the type of data and its distribution.*

|  | **Entire cohort**  **( N = 63 )** | **Protack**  **( N = 36 )** | **Reliatack**  **( N = 27 )** | **p-value** |
| --- | --- | --- | --- | --- |
| **Age > 65 years, n (%)** | 23 (36.5%) | 9 (25.0%) | 14 (51.9%) | **0.037 †** |
| **Male sex, n (%)** | 36 (57.1%) | 20 (55.6%) | 16 (59.3%) | 0.802 † |
| **BMI > 30, n (%))**  **Missing (%)** | 34 (54.0%)  1 (1.6%) | 22 (61.1%)  0 (0.0%) | 12 (44.4%)  1 (3.7%) | 0.305 † |
| **Atherosclerosis, n (%)** | 13 (20.6%) | 7 (19.4%) | 6 (22.2%) | 1.000 † |
| **Diabetic, n (%)** | 10 (15.9%) | 4 (11.1%) | 6 (22.2%) | 0.303 † |
| **CKD, n (%)** | 3 (4.8%) | 1 (2.8%) | 2 (7.4%) | 0.572 † |
| **Tot Protein < 60 g/L, n (%)**  **Missing (%)** | 0 (0.0%)  12 (19.0%)) | 0 (0.0%)  6 (16.7%)) | 0 (0.0%)  6 (22.2%)) | - |
| **Ser Albumin < 34 g/L, n (%)**  **Missing (%)** | 3 (4.8%)  11 (17.5%) | 2 (5.6%)  5 (13.9%) | 1 (3.7%)  6 (22.2%) | 1.000 † |
| **Connective tissue disorder, n (%)** | 2 (3.2%) | 0 (0.0%) | 2 (7.4%) | 0.180 † |
| **Immunosuppression, n (%)** | 3 (4.8%) | 2 (5.6%) | 1 (3.7%) | 1.000 † |
| **Smoking history, n (%)**  **(N)** | 32 (50.8%) | 16 (44.4%) | 16 (59.3%) | 0.311 † |
| **Steroids, n (%)** | 0 (0.0%) | 0 (0.0%) | 0 (0.0%) | - |
| **Warfarin, n (%)** | 2 (3.2%) | 0 (0.0%) | 2 (7.4%) | 0.180 † |

Appendix 2 - Serial Quality of Life Assessment Post-Operation using Short Form 36 (SF 36)

*P-values obtained from* ∞ *T-test,* ∆ *Wilcoxon Rank Sum Test, and* † *Fisher’s Exact Test based upon the type of data and its distribution.*

|  | **Entire cohort**  **( N = 63 )** | **Protack**  **( N = 36 )** | **Reliatack**  **( N = 27 )** | **p-value** |
| --- | --- | --- | --- | --- |
| **Day30 SF 36 domain scores, median [IQR] (N)**   - Physical function - Physical health - Emotional health - Energy/fatigue - Emotional well being - Social function - Pain - General health | 55.0 [30.0-70.0] (57)  0.0 [0.0-25.0] (57)  66.7 [0.0-100.0] (56)  45.0 [26.7-60.0] (57)  68.0 [56.0-84.0] (57)  62.5 [50.0-87.5] (57)  45.0 [32.5-71.9] (57)  68.8 [42.2-81.3] (56) | 45.0 [20.0-65.0] (33)  0.0 [0.0-25.0] (33)  66.7 [0.0-100.0] (33)  40.0 [30.0-55.0] (33)  68.0 [56.0-80.0] (33)  62.5 [50.0-87.5] (33)  45.0 [22.5-57.5] (33)  68.8 [37.5-75.0] (33) | 57.5 [30.0-71.3] (24)  0.0 [0.0-62.5] (24)  66.7[16.7-100.0] (23)  45.0 [23.8-65.0] (24)  66.0 [54.0-87.0] (24)  68.8 [50.0-87.5] (24)  45.0 [32.5-71.9] (24)  75.0 [62.5-87.5] (23) | 0.187 ∆  0.223 ∆  0.944 ∆  0.846 ∆  0.987 ∆  0.756 ∆  0.306 ∆  0.097 ∆ |
| **Day365 SF 36 domain scores, median [IQR] (N)**   - Physical function - Physical health - Emotional health - Energy/fatigue - Emotional well being - Social function - Pain - General health | 71.3 [45.3-82.2] (52)  50.0 [0.0-100.0] (52)  100.0 [33.3-100.0] (50)  54.2 [38.8-71.3] (52)  68.0[52.0-84.0] (52)  75.0 [50.0-100.0] (51)  67.5 [45.0-90.0] (52)  56.3 [37.5-68.8] (53) | 72.7 [50.1-80.2] (30)  62.5 [6.3-100.0] (30)  100.0 [66.7-100.0] (28)  55.0 [45.0-70.0] (29)  68.0 [52.0-88.0] (29)  87.5 [59.4-100.0] (28)  63.8 [37.5-90.0] (30)  50.0 [26.6-68.8] (30) | 69.6 [45.1-84.0 (22)  50.0 [0.0-100.0] (22)  100.0 [33.3-100.0] (22)  45.0 [35.0-72.5] (23)  68.0 [56.0-78.0] (23)  75.0 [50.0-100.0] (23)  67.5 [45.6-77.5] (22)  56.3 [37.5-71.9] (23) | 0.970 ∆  0.667 ∆  0.710 ∆  0.375 ∆  0.832 ∆  0.293 ∆  0.794 ∆  0.449 ∆ |

Appendix 3 - ‘VAS Pain Scores’ for Entire Cohort at all Trial Timepoints (Day)

*Boxplots showing median (bold line), interquartile range (box), values within 1.5x IQR (whiskers) and outliers (dots).*

**
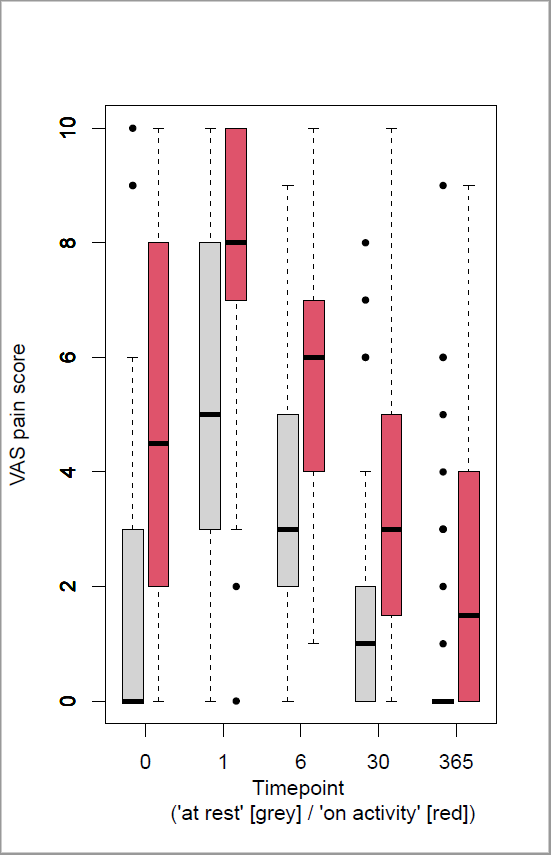
**

Appendix 4 - Median SF-36 scores for Entire Cohort on serial assessment throughout the TACKoMesh RCT.

*Colour coding of individual domains outlined in key below the x axis.*


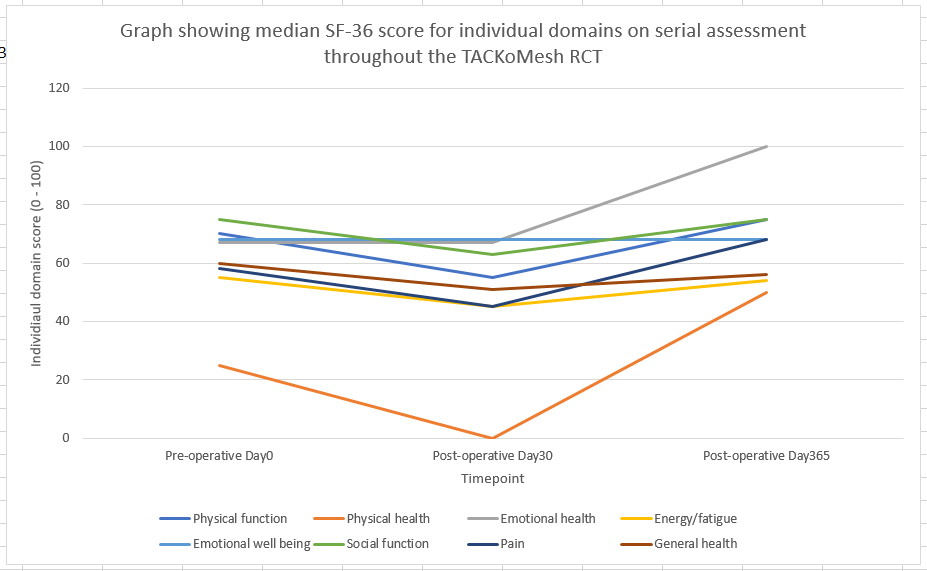


Appendix 5 (part 1) - Patient-Reported Outcomes from Post-Operative Day 30.

*P-values obtained from* ∞ *T-test,* ∆ *Wilcoxon Rank Sum Test, and* † *Fisher’s Exact Test based upon the type of data and its distribution.*

|  | **Entire cohort**  **( N = 63 )** | **Protack**  **(N = 36 )** | **Reliatack**  **(N = 27 )** | **p-value** |
| --- | --- | --- | --- | --- |
| **“Since your discharge did you experience a lot of pain?”** Yes response, n (%)  Missing (%) | 45 (71.4%)  3 (4.8%) | 27 (75.05%)  2 (5.6%) | 18 (66.7%)  1 (3.5%) | 0.386 † |
| **If Yes, how would you rate the pain,** n (%)   - Mild to moderate - Moderate to severe - Severe to unbearable | 15 (33.3%)  24 (53.3%)  6 (13.3%) | 9 (33.3%)  14 (51.9%)  4 (14.8%) | 6 (33.3%)  10 (55.6%)  2 (11.1%) | 1.000 † |
| **How many days before you could drive?,** n (%)   - 1-2 days - 3-7 days - 1-2 weeks - 2-3 weeks - > 3 weeks   (N) | 2 (5.0%)  4 (10.0%)  10 (25.0%)  7 (17.5%)  17 (42.5%)  (40) | 1 (4.6%)  1 (4.6%)  7 (31.8%)  3 (13.6%)  10 (45.5%)  (22) | 1 (5.6%)  3 (16.7%)  3 (16.7%)  4 (22.2%)  7 (38.9%)  (18) | 0.595 † |
| **How many days before you could cook or clean?,** n (%)   - 1-2 days - 3-7 days - 1-2 weeks - 2-3 weeks - > 3 weeks   (N) | 8 (14.8%)  12 (22.2%)  12 (22.2%)  10 (18.5%)  12 (22.2%)  (54) | 2 (6.7%)  6 (20.0%)  9 (30.0%)  5 (16.7%)  8 (26.7%)  (30) | 6 (25.0%)  6 (25.0%)  3 (12.5%)  5 (20.8%)  4 (16.7%)  (24) | 0.245 † |
| **How many days before you could walk without painkillers?,** n (%)   - 1-2 days - 3-7 days - 1-2 weeks - 2-3 weeks - > 3 weeks   (N) | 7 (13.2%)  14 (26.4%)  13 (24.5%)  9 (17.0%)  10 (18.8%)  (53) | 3 (9.7%)  9 (29.0%)  7 (22.6%)  5 (16.1%)  7 (22.6%)  (31) | 4 (18.2%)  5 (22.7%)  6 (27.3%)  4 (18.2%)  3 (13.6%)  (22) | 0.822 † |

Appendix 5 (part 2) - Patient-Reported Outcomes from Post-Operative Day 30.

*P-values obtained from* ∞ *T-test,* ∆ *Wilcoxon Rank Sum Test, and* † *Fisher’s Exact Test based upon the type of data and its distribution.*

|  | **Entire cohort**  **( N = 63 )** | **Protack**  **( N = 36 )** | **Reliatack**  **( N = 27 )** | **p-value** |
| --- | --- | --- | --- | --- |
| **How many days before you returned to work?** (n (%))   - 1-2 days - 3-7 days - 1-2 weeks - 2-3 weeks - > 3 weeks   (N) | 1 (3.9%)  0  1 (3.9%)  2 (7.7%)  22 (84.6%)  (26) | 1 (5.0%)  0  0  2 (10.0%)  17 (85.0%)  (20) | 0  0  1 (16.7%)  0  5 (83.3%)  (6) | 0.447 † |
| **How many days before you stopped taking pain killers?** (n (%))   - 1-2 days - 3-7 days - 1-2 weeks - 2-3 weeks - > 3 weeks   (N) | 5 (10.2%)  13 (26.5%)  4 (8.2%)  12 (24.5%)  15 (30.6%)  (49) | 1 (3.6%)  8 (28.6%)  1 (3.6%)  9 (32.1%)  9 (32.1%)  (28) | 4 (19.1%)  5 (23.8%)  3 (14.3%)  3 (14.3%)  6 (28.6%)  (21) | 0.206 † |
| **How long until you were fully recovered?** (n (%))   - 1-2 days - 3-7 days - 1-2 weeks - 2-3 weeks - > 3 weeks   (N) | 1 (2.0%)  1 (2.0%)  4 (7.8%)  5 (9.8%)  40 (78.4%)  (51) | 0  0  3 (10.0%)  3 (10.0%)  24 (80.0%)  (30) | 1 (4.8%)  1 (4.8%)  1 (4.8%)  2 (9.5%)  16 (76.2%)  (21) | 0.623 † |
